# Transitional care for patients with acute stroke—A priority‐setting project

**DOI:** 10.1111/hex.13517

**Published:** 2022-05-02

**Authors:** Liss Marita Solbakken, Birgitta Langhammer, Antje Sundseth, Therese Brovold

**Affiliations:** ^1^ Department of Physiotherapy Oslo Metropolitan University Oslo Norway; ^2^ Department of Neurology Akershus University Hospital Lørenskog Norway

**Keywords:** collaboration, communication, priority setting, research needs, stroke, transitional care, user involvement

## Abstract

**Background:**

The scope of this priority‐setting process is communication and collaboration in transitional care for patients with acute stroke. Actively involving persons with stroke and their family caregivers is important both in transitional care and when setting priorities for research. Established priority‐setting methods are time‐consuming and require extensive resources. They are therefore not feasible in small‐scale research. This article describes a pragmatic priority‐setting process to identify a prioritized top 10 list of research needs regarding transitional care for patients with acute stroke.

**Methods:**

A pragmatic priority‐setting approach inspired by the James Lind Alliance was developed. It involves establishing a user group, identifying the research needs through an online survey, analysing and checking the research needs against systematic reviews, culminating in an online prioritization of the top 10 list.

**Results:**

The process was completed in 7 months. A total of 122 patients, family caregivers, health personnel and caseworkers submitted 484 research needs, and 19 users prioritized the top 10 list. The list includes the categories ‘patients and caregivers’ needs and health literacy’, ‘health personnel's common understanding’, ‘information flow between health personnel and patients and caregivers’, ‘available interventions and follow‐up of patients and caregivers’, ‘interaction and collaboration between health personnel and caseworkers across hospital and primary healthcare’ and ‘disabilities after stroke’.

**Conclusion:**

This paper outlines a pragmatic approach to identifying and prioritizing users' research needs that was completed in 7 months. The top 10 list resulting from this priority setting process can guide future research relating to communication and collaboration during the transition from hospital to the community for patients with stroke.

**Patient and Public Contribution:**

Members of three stroke organizations participated in the advisory group. They gave feedback on the scope and the process, distributed the surveys and prioritized the top 10 list. Persons with stroke and their caregivers submitted research needs in the survey.

## 1 INTRODUCTION

Depending on functional and cognitive disabilities, patients may need long‐term rehabilitation after discharge from the hospital to ensure the best possible outcome.^1^, ^2^, ^3^ The concept of transitional care includes both hospital and posthospital interventions that promote a safe and timely transfer of patients between levels of care.^4^, ^5^, ^6^ However, while there is no consensus on what constitutes transitional care after stroke, engaging both patients and healthcare personnel is essential, and interventions may include discharge planning, patient and family education, follow‐up care, rehabilitation and the transfer of information between care providers.^4^, ^6^, ^7^, ^8^, ^9^

Healthcare reforms in Norway have led to more responsibility for rehabilitation being transferred from hospitals to primary healthcare (PHC) in the municipalities,^10^ resulting in earlier discharge from hospital.^11^ Lack of involvement and communication, for patients in need of follow‐up, can lead to uncertainty and a feeling of lack of control.^12^ After hospital discharge, patients may experience a variety of unmet needs, including needs for more service, information and therapy,^13^, ^14^ indicating that there is insufficient communication between the patient and health personnel, as well as between health personnel across care levels.

There is an increasing focus on patients' and users' rights to participate in research, and legal and health policy documents support these processes.^15^, ^16^, ^17^ Inviting users to set research priorities can reduce the described mismatch between patients' needs and researchers' agendas,^18^ and reduce research waste.^19^ In addition, since users may feel a greater sense of ownership of the research and its results, this could have an impact on the implementation of the research results, as the research is perceived as more relevant.^20^, ^21^ Priority setting processes focus on involving users to identify their research needs and to prioritize these needs.^18^, ^22^, ^23^ There are few publications dealing with priority setting processes in the stroke context, and none on transitional care from hospital to PHC. Two large priority setting processes in the United Kingdom resulted in four top 10 lists: Pollock et al.^24^ prioritized a top 10 list relating to life after stroke, Franklin et al.^25^ a top 10 list for people with aphasia, while the Stroke Association prioritized two 10 top lists relating to stroke prevention, diagnosis, prehospital and hospital care and stroke rehabilitation and long‐term care.^26^

There is no gold standard method for setting priorities for research.^21^, ^27^ Because well‐described priority setting approaches require resources, are time‐consuming and often aim to set priorities at the national level,^23^, ^27^ they might need to be adapted to fit smaller research projects.^21^ The aim of this article is therefore to describe the pragmatic priority setting process used to identify a prioritized top 10 list of research needs regarding communication and collaboration in transitional care for patients with acute stroke (TracStroke).

## 2 METHODS

The TracStroke project is part of the Bridge Builder Initiative at Oslo Metropolitan University^28^ and is affiliated with Akershus University Hospital. The university hospital has the largest stroke unit in Norway, serving 24 municipalities with approximately 560,000 inhabitants.^29^

The TracStroke project is based on the Bridge‐Building Model developed at the university,^30^ which entails developing needs‐led research approaches. The first author has attended a needs‐led research course at the university, while the last author has previously participated in needs‐led projects. The scope of the TracStroke project is examined in a Norwegian context, where increased responsibility for patients' care and rehabilitation was transferred to the municipalities through the Coordination Reform of 2012.^10^ The scope is derived from a clinical question raised by clinicians at the hospital's stroke unit, as recommended in the Bridge Building Model.^30^ As preparations are recommended when developing an approach,^20^ the first author contacted health personnel and organizations for persons with stroke and requested their feedback on whether the scope of research needs relating to communication and collaboration in transitional care for patients with stroke was interesting and whether they wished to participate in a priority‐setting process. Since they responded positively, the project's scope was defined as ‘communication and collaboration in the service transition from hospital to municipal rehabilitation after stroke’.

**Figure 1:**
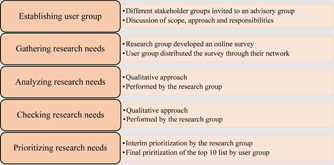
TracStroke priority‐setting approach.

The method used for priority setting in the TracStroke project was strongly inspired by the James Lind Alliance (JLA) Guidebook, which explains the methods and principles of its priority‐setting partnership (PSP),^23^ as well as by frameworks described in other needs‐led research.^20^, ^31^ The JLA is a systematic process in which patients, caregivers and health professionals are invited to take part in an equal PSP to identify and prioritize research needs.^23^ The process includes appointing a steering group and establishing a PSP and identifying the users' research needs, before processing and checking them against systematic reviews. The final step is a prioritization process that includes both an interim process and a workshop to arrive at a top 10 list of prioritized research needs. The JLA is a well‐known method,^32^ but it requires resources and supervision by a JLA consultant^23^ and is time‐consuming.^27^ This means that it was not feasible for the TracStroke project and the research group to use. Our approach did not include the roles and responsibilities of the JLA resources or the steering group,^23^ and the research group carried out and led the project. During the development of the approach, the focus was on creating a pragmatic and feasible approach to identify users' research needs within the timeframe and limited resources available. Users were broadly defined to include persons with stroke, their caregivers and health personnel all of whom could be affected by the research.^16^, ^17^ The research group consisted of the authors of this article. As several authors encourage describing the approach used^33^, ^34^ and the use of checklists to ensure the quality and transparency of the approach,^35^, ^36^ the steps in the TracStroke priority‐setting approach (Figure 1) will be described in this section.

### 2.1 Establishing the user group

The stakeholders in this process were broadly defined to include patients with stroke, their caregivers and various health personnel. Persons with stroke and caregivers were important as they experience the outcomes of a stroke, and their experiences are important when developing research that might meet their needs. Health personnel in both the hospital and PHC, and caseworkers in the municipalities were included since they communicate, cooperate and coordinate their activities to make sure patients receive appropriate care after discharge from the hospital. Unlike the JLA PSP, where the steering groups work closely with the researchers,^23^ the user group had an advisory role and was kept informed and consulted throughout the process. The user group's main tasks were to give feedback and advise about the scope and the priority‐setting process, and promote and participate in the online surveys for gathering and prioritizing research needs.

As the priority‐setting process entailed meetings, members were recruited from neurologic and allied health units at the university hospital, the municipalities around the hospital and the local branches of the three organizations for persons with stroke. The invitation included information about the scope and purpose of the project, and that their involvement entailed an advisory role. They were also informed that meetings would take place at the hospital, and that refreshments would be provided and travel expenses refunded. At the first and only meeting with the user group, the scope and priority‐setting approach were presented and discussed. The group was encouraged to discuss the different concepts and terms used to describe the scope and the approach, their relevance, and other aspects that triggered engagement. A consensus was reached on the scope, the members' involvement and responsibilities in relation to gathering and prioritizing research needs.

### 2.2 Gathering research needs

To identify the research needs of patients, caregivers, health personnel and caseworkers, the research group developed an online survey.^23^ The questions were phrased differently for persons with stroke and their caregivers, and health personnel and caseworkers to reflect on their specific experiences in the transition (Table 1). The user group piloted the survey and was asked to comment on the questions, their wording and user‐friendliness. There were no limitations on how many inputs each respondent could provide.

**Table 1:** Questions for identifying research needs

*Persons with stroke and caregivers*
What do you think is important to investigate regarding communication between health personnel and patients and caregivers? What do you think should be given most focus upon discharge from hospital? What do you think the PHC should focus most on after discharge from hospital? Are there other aspects of discharge from hospital to PHC that it is important to investigate?
*Health personnel*
What do you think is important to investigate regarding communication between health personnel in the hospital and PHC? What do you think is important to investigate regarding communication between health personnel and patients and caregivers? What do you think is important to investigate regarding collaboration between health personnel in the hospital and PHC? Are there other aspects of discharge from hospital to PHC that it is important to investigate?
*Caseworkers*
What do you think is important to investigate regarding communication between health personnel in the hospital and PHC? What do you think is important to investigate regarding collaboration between health personnel in the hospital and PHC? Are there other aspects of discharge from hospital to PHC that it is important to investigate?

Abbreviation: PHC, primary healthcare.

Participants were recruited using convenience/snowballing sampling to reach as many as possible within the timeframe. The survey was promoted by the user group and the hospital through social media and newsletters, and through patient and health‐related networks.

### 2.3 Analysing research needs

Before the analysis, the data set was managed in Microsoft Excel to organize and group research needs and to ensure that each respondent group's (patients, caregivers, health personnel in hospital, health personnel in PHC, caseworkers) research needs would be apparent as recommended by the JLA.^23^ The submitted research needs were organized under the survey question categories: information, collaboration and other aspects of discharge.

As JLA recommends categorizing and grouping similar research needs,^23^ the qualitative analysis of the submitted research needs was inspired by thematic analysis.^37^ The first step in the analysis entailed reading the research needs several times to get an impression of the material and to identify preliminary categories that capture the submitted research need. The second step involved identifying, sorting and coding research needs within and across categories. Each category was sorted into subgroups that revealed different aspects of the main category. Research needs or comments that were not related to transitional care challenges were removed during Steps 1 and 2. In the third step, the research needs in each subgroup were summarized into one or more indicative questions. The questions were based on the research needs submitted as questions, and the research needs submitted as comments were categorized under a similar research question. In subgroups without a formulated question, an indicative question was formulated based on the submitted input. In the last step, similar research needs questions were combined with rephrased questions. The JLA suggests combining and rephrasing similar questions,^23^ which is both an interpretative and a pragmatic process that makes the list to be checked against the evidence base shorter and more manageable (Table 2).

**Table 2:** Development of categories and questions

Categories	Coded research need	Examples of the research questions
Follow‐up	What kind of follow‐up do patients receive after discharge from the stroke unit?	What kind of follow‐up do patients receive after discharge from the stroke unit?
	What kind of follow‐up do complex patients receive after discharge from the stroke unit?	
	What is done to ensure patients receive the right follow‐up after discharge?	
Information	What kind of information do patients and their families receive during stroke follow‐up?	How do health personnel inform the patients about stroke and what to expect?
	How is the information adapted to the patient's information needs?	
	How is the patient informed and guided during and after the hospital stay?	
Disabilities	How to communicate with patients with different kinds of cognitive needs.	How do health personnel adapt their communication for patients with cognitive needs?
	How to communicate with patients with aphasia.	
Patient and caregiver needs	Do the patients understand the information they are given?	How do patients experience the information given by health personnel?
	How do the patients perceive the information given to them?	
Collaboration	How can the hospital and the community collaborate to make each other better?	How do health personnel cooperate across levels of care and how can the collaboration be improved?
	Is there any collaboration and how can it be improved?	
	How to prioritize collaboration in a hectic work situation.	
Common understanding	How knowledgeable is the hospital about the healthcare services in the community?	How knowledgeable are health personnel about services across care levels?
	How to increase the understanding of how we contribute	
	How to create a common understanding of follow‐up and the services available	

### 2.4 Checking research needs

After the analysis, to prevent duplicate research, the list of research needs was checked against systematic reviews to verify whether there were true knowledge gaps.^23^ Questions that had been addressed and answered by systematic reviews were removed from the list, while relevant research recommendations from the reviews were added to the list.^23^ Systematic searches were conducted in the databases Cochrane, Epistemonikos, Medline and PsycINFO. The search strategy used the MeSH terms and equivalent text words: ‘stroke’ AND ‘discharge’ OR ‘transition’ OR ‘transfer’ for all databases, and, in Medline and PsycINFO, the additional MeSH terms: AND ‘hospital’ AND ‘home health care’ were used. Systematic reviews published in peer‐reviewed journals after 2010 were included. The abstracts of the systematic reviews were read to check whether the research needs categories were mentioned as outcomes or categories for the review.

The analysis and checking of research needs were performed by the first author and checked and discussed by the research group. The user group received a PowerPoint presentation, with an audio explanation of the analysis in an email, and was encouraged to ask questions if anything was unclear and to give feedback.

### 2.5 Prioritizing research needs

Due to the COVID‐19 restrictions in Norway, which resulted in a national lockdown, the prioritization was carried out in two steps and did not include workshops as recommended in JLA (Figure 1). Before starting the prioritization, a new round of combining similar questions within and across the categories was performed, creating a long list for prioritization. In addition, the research group considered whether the research questions were feasible for research purposes. The interim prioritization was conducted by the research group. The research needs represented by at least three of the six respondent groups (patients, caregivers, health personnel hospital, health personnel PHC, caseworkers, reviews) were prioritized in a shortlist for final prioritization by the users. The user group was informed about this process through a PowerPoint presentation distributed by email.

The final prioritization included an anonymous electronic survey conducted to prioritize a shortlist for a top 10 list. The user group received a link to the survey, and the members distributed it within their networks to include more patients and health personnel, as well as caregivers and caseworkers. Those participating were asked to select the 10 most important research needs questions from the shortlist. The research needs questions with most points constituted the top 10 list. The user group received a final presentation with the top 10 list and the proposed research question for the TracStroke project.

### 2.6 Ethical considerations

In Norway, needs‐led research does not require approval by the Regional Committees for Medical and Health Research. The study was discussed with the Norwegian Centre for Research Data and, since the survey did not gather personal information and contained informed consent information, it did not require their approval either. The user group received and signed a written informed consent at the first meeting. The survey contained informed consent information in accordance with the guidelines set out by the Norwegian Centre for Research Data. The first page of the survey contained information outlining the purpose of the study and its anonymous nature and asked participants to refrain from writing confidential information. Participants were informed that their participation was voluntary and that by answering the survey they gave their consent.

## 3 RESULTS

The TracStroke priority‐setting process lasted from September 2019 until April 2020.

### 3.1 The user group

The user group consisted of 12 members (Table 3). The three regional stroke organizations were invited to appoint a member each. The hospital appointed two nurses, one neurologist, one physiotherapist and one consultant. Two physiotherapists, one occupational therapist and a physician represented the municipalities.

**Table 3:** Description of the user group

Stakeholder groups	Profession	Gender
Patient organizations	Three persons with stroke	Two men/one woman
Hospital	Two nurses One neurologist One physiotherapist One consultant	Five women
Primary healthcare	Two physiotherapists One occupational therapist One physician	One man/three women

### 3.2 Participation in the online survey

**Figure 2:**
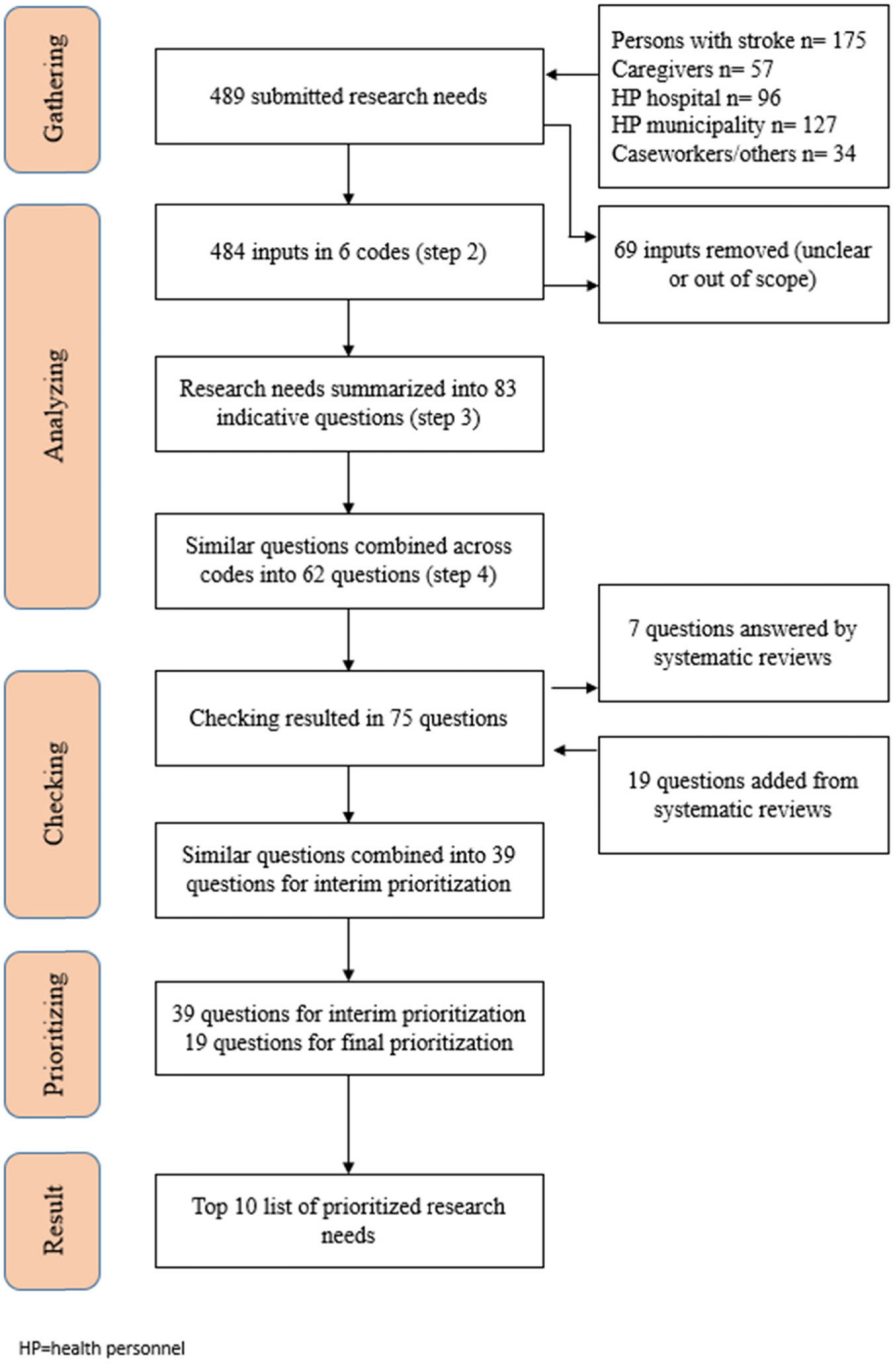
Flow‐chart of the TracStroke process.

The survey gathered research needs from 122 patients, caregivers, health personnel and caseworkers through the online survey (Table 4). The majority of the 489 submitted inputs came from patients (175), while their caregivers submitted 57 research needs (Figure 2). Health personnel working in PHC submitted 127 research needs, while health personnel from hospitals submitted 96 and caseworkers and others submitted 34. Nearly 60% of health personnel were either physiotherapists (32%) or nurses (27%), while 15% were occupational therapists, 12% physicians and the rest other healthcare personnel.

**Table 4:** Respondents in the online survey and prioritization

Respondents	Survey	Prioritization
Persons with stroke	40	5
Caregivers	13	2
Health personnel hospital	24	5
Health personnel primary healthcare	35	3
Caseworkers/others	10	4
*Total*	*122*	*19*

### 3.3 Categorization of the research needs

Six categories were identified in the thematic analysis (Table 2). The categories ‘Collaboration’ and ‘Follow‐Up’ were identified in 50% of the submitted research needs (Table 5). Within the category ‘Collaboration’, 85% of the research needs were evenly divided by health personnel and patients, while, within the category ‘Follow‐Up’, 63% of the input was submitted by patients (Table 6). An even distribution of patients, caregivers and health personnel submitted research needs relating to the category ‘Patient's & Caregiver's Needs’, while 76% of the research needs relating to the category ‘Common Understanding’ were submitted by health personnel, and patients and caregivers submitted 81% of the needs relating to ‘Disabilities after stroke’. Table 7 shows how the different respondent groups' research needs are distributed across different categories.

**Table 5:** Distribution of the 484 research needs across categories and respondents

	Information	Follow‐up	Collaboration	P&C needs	Common understanding	Disabilities	No. of RN
P	4.5%	15.7%	6.6%	4.3%	2%	3.9%	171
C	1.6%	3.7%	2.6%	3%	0	0.4%	56
HPH	3.9%	1.8%	7%	4.3%	2.2%	0.4%	96
HPP	6.6%	2.6%	8.2%	3.9%	4.3%	0.6%	128
CW	1.6%	0.8%	1.4%	1%	1.8%		33
%	18.2%	24.6%	25.8%	16.5%	10.3%	5.3%	100%/484

Abbreviations: CW, caseworkers; HPH, health personnel hospital; HPP, health personnel PHC; P, persons with stroke; RN, research needs.

**Table 6:** Respondent representation within categories

	Information	Follow‐up	Collaboration	P&C needs	Common understanding	Disabilities
No of RN	89	120	126	81	42	26
P	24.7%	63.3%	25.4%	25.9%	2.4%	73.1%
Caregivers	9%	15%	10.3%	18.5%	0	7.7%
HPH	21.3%	7.5%	27%	25.9%	26.2%	7.7%
HPP	36%	10.8%	31.7%	23.5%	50%	11.5%
CW	9%	3.3%	5.6%	6.2%	21.4%	0
%	100%	99.9%	100%	100%	100%	100%

Abbreviations: CW, caseworkers; HPH, health personnel hospital; HPP, health personnel PHC; P, persons with stroke; RN, research needs.

**Table 7:** Respondents' research needs across categories

	Information	Follow‐up	Collaboration	P&C needs	Common understanding	Disabilities	%
P	12.9%	44.4%	18.7%	12.3%	0.6%	11.1%	100%
C	14.3%	32.1%	23.2%	26.8%	0	3.6%	100%
HPH	19.8%	9.4%	35.4%	21.9%	11.4%	2.1%	100%
HPP	25%	10.2%	31.3%	14.8%	16.4%	2.3%	100%
CW	24.2%	12.1%	21.2%	15.2%	27.3%		100%

Abbreviations: CW, caseworkers; HPH, health personnel hospital; HPP, health personnel PHC; P, persons with stroke; RN, research needs.

### 3.4 Checking the research needs

The systematic search identified 26 relevant systematic reviews. The 62 research needs were checked against the systematic reviews. Twelve of the reviews answered seven research needs, six gave rise to nineteen questions, while eight did neither (Figure 2). The systematic reviews' contributions to the research needs were: information and guidance of patients and their families,^9^, ^38^, ^39^ how health personnel communicates and cooperate with each other,^40^, ^41^, ^42^, ^43^ and how follow‐up is offered and delivered to patients and their caregivers.^9^, ^44^, ^45^, ^46^ After checking and adding research needs from the reviews, the research group combined similar research needs, resulting in a list of 39 research needs for prioritization.

### 3.5 The prioritaztion of the top 10 list

In the interim prioritization, the research group produced a shortlist of 19 questions that were represented by at least three of the six respondent groups (patients, caregivers, health personnel in hospital, health personnel in PHC, caseworkers, systematic reviews).

In the final prioritization, 19 users (Table 4) participated in voting for the top 10 list of research needs (Table 8). The two research needs at the top of the list received 74% of the votes, and input from patients, caregivers and health personnel contributed to these questions. The two lowest priority research needs received 53% of the votes. Question number 4 is based on input from all the user groups (Table 8).

**Table 8:** Top 10 list of research needs

Votes	Categories	Research needs	Respondent
14	Disabilities	How do health personnel adapt their communication for patients with cognitive needs?	1,2,3,4
14	Follow‐up	What kind of follow‐up do patients receive after discharge from the stroke unit?	1,2,3,4
13	P&C needs	How do patients experience the information given to them by health personnel?	1,3,5
12	Information	How do health personnel communicate with patients and their caregivers about stroke and the need for a follow‐up?	1,2,3,4,5,6
12	P&C needs	How are the patient's caregivers cared for?	1,2,3
11	Collaboration	How do health personnel cooperate across levels of care and how can collaboration be improved?	1,3,4,6,
11	P&C needs	How do patients and caregivers experience the collaboration between the hospital and PHC?	1,2,3,4,5
10	P&C needs	What are the patients' and their caregivers' expectations of the PHC services?	1,2,3,4,5
10	Common understanding	How knowledgeable are health personnel about services across care levels?	3,4,5,6	
10	Common understanding	How well do health personnel across care levels understand each other when communicating?	1,3,4,5,6	

Abbreviations: 1, persons with stroke; 2, caregivers; 3, health personnel hospital; 4, health personnel PHC; 5, caseworkers; 6, reviews.

## 4 DISCUSSION

This article describes a pragmatic priority‐setting process aimed at identifying a prioritized top 10 list of research needs relating to communication and collaboration in transitional care for patients with acute stroke. In this process, 122 patients, their caregivers, health personnel and caseworkers submitted their research needs, while 19 users prioritized the top 10 list of research needs. The list includes four questions regarding ‘patients' and caregivers' needs and health literacy’ and two questions regarding ‘health personnel's common understanding’. The categories ‘information flow between health personnel and patients and caregivers’, ‘available interventions and follow‐up for patients and caregivers’, ‘interaction and collaboration between health personnel and caseworkers across the hospital and PHC’ and ‘disabilities after stroke’ are represented with one question each in the top 10 list.

Every main category from the analysis is represented in the top 10 list. The categories are similar to the most common categories in other priority setting studies, including ‘patients’, ‘health care professionals’, ‘carers’, ‘health care system’ and ‘treatment’.^32^ This could indicate that, even though our approach is less rigorous than the JLA, it was conducted rigorously enough to embrace important categories. We included research needs phrased as comments and not just questions and, since patients' and caregivers' submissions are more often rejected,^22^ this might have increased their impact on the development of the categories.

The TracStroke top 10 list complements the other priority‐setting processes relating to stroke. Whereas Pollock et al.,^24^ Franklin et al.^25^ and the Stroke Association^26^ present top 10 lists concerning prehospital and hospital care and rehabilitation, our top 10 list concerns communication and collaboration during the transition from hospital to the community. The questions in the top 10 list are mostly phrased to suit qualitative research. The different foci of the top 10 lists can be affected by their different scopes and how the questions were phrased in the studies. We specifically asked about communication and collaboration relating to the discharge process and rehabilitation. Pollock et al.,^24^ on the other hand, asked about stroke treatment uncertainties, which lead to questions concerning interventions. Asking specifically after ‘treatment uncertainties’ can be misunderstood as referring to medical treatment, and therefore have an impact on what kind of questions are raised by those submitting their research needs and on the inclusion of submitted research needs.^22^

The scope of TracStroke was narrowed down to information and collaboration, but we included research needs relating to the categories ‘follow‐up’ and ‘disabilities after stroke’ since patients and caregivers submitted these needs. Most of the research needs within the categories ‘available interventions and follow‐up for patients and caregivers’ and ‘disabilities after stroke’ were submitted by patients and caregivers. This might reflect patients' and their caregivers' need for more predictable information and follow‐up after discharge, as patients and caregivers describe a stroke as a profound disruption of life and need support to rebuild their lives and come to terms with the new reality.^47^

The top four questions in our top 10 list indicate that there are knowledge gaps regarding the information given to patients about stroke, how it affects them and what they can expect when they return home after discharge from the hospital. Questions about available interventions and follow‐up could depend on the health system. In Norway, the length of hospital stays has decreased due to various healthcare reforms, whereby more responsibility for acute and subacute rehabilitation has been transferred to the municipalities.^11^ The difference in the PHC resources,^11^ the patients' resources^48^ and knowledge of the services available in the different communities, might also influence the care and follow‐up the patients receive after hospital discharge. Therefore, different contexts can yield different answers to these questions. Actively involving patients and their caregivers when providing information they need is important in transitional care^4^, ^8^ and to improve their knowledge about stroke.^49^ More research on active interventions to provide information to patients with stroke is encouraged by a Cochrane review.^49^

Unsurprisingly, since discharge planning is an integral part of transitional care,^4^, ^5^, ^6^ most of the research needs within the categories ‘interaction and collaboration between health personnel and caseworkers across the hospital and PHC’ and ‘health personnel's common understanding’ were submitted by health personnel in the hospital and PHC groups (Table 6). Within the health personnel group, a high percentage of the research needs also included patients' and caregivers' needs, and information for patients and their caregivers (Table 7). The TracStroke top 10 list is in line with other studies with categories focusing on patients' and caregivers' needs, as well as health personnel's communication and collaboration on patients' needs.^32^ A study across five European countries describes the hospital—PHC interface as fragmented due to ‘the inward focus of hospital care providers, lack of awareness to needs, skills and work patterns of professional counterparts, lack of collaborative attitudes and the relationship between the hospital and primary care providers’.^50^ One of our research needs, ‘How do health personnel cooperate across levels of care and how can collaboration be improved?’, calls for answers to the issue of collaboration. Lindblom et al.'s^12^ study shows a communication that is sensitive to different situations and also the contexts between care levels, which can facilitate collaboration and a common understanding of the discharge process. Knowledge about each other's competencies and routines seems to be important for building trust in each other's assessment and decisions about the patient's rehabilitation needs. More studies on communication and collaboration across care levels and knowledge of the services available to patients could generate knowledge about how to increase actual collaboration and patient‐centred rehabilitation.

Recruiting user group members through open invitations to the municipalities was more challenging than expected. Although they did not specify why they could not participate, we can speculate. One reason could be that the invitation or scope was not interesting enough, although this is contradicted by the fact that 37% of the respondents in the survey were from the PHC sector. Furthermore, the PHC might not have the time or resources to participate. Following the implementation of the Coordination Reform in Norway, PHC health personnel have experienced that an increasing number of frail patients are discharged into their care without more resources being allocated to manage the increased workload and responsibility.^11^ Lastly, the willingness and resources required to prioritize research may differ between hospitals and PHC. There is an increased focus on research in the municipalities, as reported in both political and research council documents,^16^, ^51^ but there are no studies reporting PHC personnel's willingness to be, or experience of being, involved in research, and more research is needed in these areas.^52^

### 4.1 Strengths and limitations

In the process of identifying research needs, we have followed the JLA, but have made some pragmatic adjustments to the approach. The TracStroke approach did not include a JLA consultant nor a steering group, and these roles were filled by the research group. By assigning these responsibilities to the research group, we could save both time and costs, although this affected the level of involvement compared to a JLA PSP. Involving the user group in the analysis was not planned for as this requires specific competencies.^23^ We could have involved the users in the interim prioritization through an online survey. This could have changed the outcome list for prioritization. However, the interim prioritization ensured that the research questions for final prioritization were broadly supported by the different respondent groups, and it also saved us time.

We aimed for and, in part, achieved a broad composition of participants in the user group. Unfortunately, caregivers and caseworkers from the municipalities were not represented and more effort should have been made to include these groups. We believe that the user group felt ownership of the project since it promoted and distributed the survey through different media and reached the different stakeholder groups. The research needs gathered through the survey added new perspectives to the research group. Hopefully, this participation will contribute to research being more relevant and to it meeting the needs of those who will benefit from and implement the research.^20^ Although the survey resulted in broad participation from different user groups, we did not gather any demographic information. Online surveys are unlikely to reach the voices of those in low socioeconomic groups, those with the greatest unmet needs, or those with low health and/or online literacy.^53^ These voices tend to be less represented in research. Face to face meetings in the communities could be more suited to reach these groups.^54^

The COVID‐19 restrictions and national lockdown affected both the user group meetings and the prioritization workshop that was planned. Instead of meetings, the user group received a written summary of the different steps. Unfortunately, this led to less feedback and discussions with the user group. A smaller group might have worked more closely with the research group and could have resulted in more involvement and sharing of knowledge. The online prioritization, although group discussions and agreement were lacking, included more and broader user participation than the user group (Table 4). In addition, the online survey may have been a positive feature as the participants could prioritize their own opinions, without being influenced by those with strong voices.^23^ Online prioritization could also have been used for the interim prioritization, which could have affected the final list for prioritization. Following the pandemic, JLA has described how workshops can be adapted to an online setting.^55^ An online conference was not considered feasible to hold due to the strain on the healthcare personnel during the pandemic and a lack of facilities, such as laptops, computers and space to participate.

One strength of the TracStroke process is its qualitative approach, which includes input not written as questions. This strengthened the patient's voice. Our analysis shows that, out of the 161 research needs submitted as a question, only 12.5% are formulated by patients and caregivers. If only formulated questions were included, 91% of the patients' and caregivers' research needs would be excluded. Including their comments in the analysis meant that they were represented in the indicative research questions. This may have contributed to broader categories and subgroups since patients and caregivers have other research needs than clinicians and researchers.^18^, ^56^ The way in which similar research needs were combined might have been affected by how the questions were phrased. Since the patients' and caregivers' research needs often were phrased as comments, those phrased by health personnel might have been preferred, although this was not investigated.

Although systematic searches were performed to check whether research needs were in fact knowledge gaps, it is a limitation, in that these searches were based on the terms related to ‘stroke’, ‘discharge’ and ‘transition’ and were not exhaustive for each specific research need, as JLA advises. Therefore, some of the research needs in the top 10 list might already be answered.

## 5 CONCLUSION

This article outlines a pragmatic approach to identifying research needs and setting priorities for research. The adaptions to the JLA PSP made it possible to complete the priority setting within the timeframe of 7 months, and the TracStroke approach might therefore also be feasible for other research projects. The result of the process is a prioritized top 10 list of research needs relating to care during the transition from a hospital stroke unit to rehabilitation in municipal healthcare, including the categories: patients' and caregivers' needs, disabilities after stroke, information flow, collaboration and common understanding among health personnel. The top 10 list will guide the development of research at the university and subsequently benefit the patients in their encounters with healthcare services after a stroke.

## CONFLICTS OF INTEREST

The authors declare no conflicts of interest.

## Data Availability

The data that support the findings of this study are available from the corresponding author upon reasonable request.

## References

[1] The Norwegian Directorate of Health . *Norwegian guideline on management and rehabilitation of stroke*; 2017. Accessed August 2021. www.helsedirektoratet.no/retningslinjer/hjerneslag

[2] Hebert D , Lindsay MP , McIntyre A , et al. Canadian stroke best practice recommendations: stroke rehabilitation practice guidelines, update 2015. Int J Stroke. 2016;11(4):459‐484. 10.1177/1747493016643553 27079654

[3] Fjærtoft H , Skogseth‐Stephani R , Indredavik B , Bjerkvik T , Varmdal T . *Norsk hjerneslagregister‐Årsrapport 2019‐Med plan for forbedringstiltak Register*. 2020. Accessed December 16, 2021.https://www.kvalitetsregistre.no/sites/default/files/1_arsrapport_2019_norsk_hjerneslagregister_justert_21.10.2020.pdf

[4] NTOCC . Transition of care measures. The National Transitions of Care Coalition. Accessed June 20, 2021. https://static1.squarespace.com/static/5d48b6eb75823b00016db708/t/5d49bc5dc8a2700001c6ac7e/1565113438113/TOC%2BMeasures.pdf

[5] Naylor MD , Keating SA . Transitional care. Am J Nurs. 2008;108(9):58‐63. 10.1097/01.NAJ.0000336420.34946.3a PMC2768550 18797231

[6] Coleman EA , Boult C . Improving the quality of transitional care for persons with complex care needs. J Am Geriatr Soc. 2003;51(4):556‐557. 10.1046/j.1532-5415.2003.51186.x 12657079

[7] Burke RE , Kripalani S , Vasilevskis EE , Schnipper JL . Moving beyond readmission penalties: creating an ideal process to improve transitional care. J Hosp Med. 2013;8(2):102‐109. 10.1002/jhm.1990 23184714 PMC3650641

[8] Puhr MI , Thompson HJ . The use of transitional care models in patients with stroke. J Neurosci Nurs. 2015;47(4):223‐234. 10.1097/JNN.0000000000000143 25906245

[9] Prvu Bettger J , Alexander KP , Dolor RJ , et al. Transitional care after hospitalization for acute stroke or myocardial infarction: a systematic review. Ann Intern Med. 2012;157(6):407‐416. 10.7326/0003-4819-157-6-201209180-00004 22986378

[10] Norwegian Ministry of Health and Care Services . The Coordination Reform—proper treatment—at the right place and right time. 2009.

[11] Gautun H , Syse A . Earlier hospital discharge: a challenge for Norwegian municipalities. Nord J Soc Res. 2017;8:1‐17 10.7577/njsr.2204

[12] Lindblom S , Ytterberg C , Elf M , Flink M . Perceptive dialogue for linking stakeholders and units during care transitions—a qualitative study of people with stroke, significant others and healthcare professionals in Sweden. Int J Integr Care. 2020;20(1):11. 10.5334/ijic.4689 PMC7101013 32256255

[13] Chen T , Zhang B , Deng Y , Fan J‐C , Zhang L , Song F . Long‐term unmet needs after stroke: systematic review of evidence from survey studies. BMJ Open. 2019;9(5):e028137. 10.1136/bmjopen-2018-028137 PMC6530326 31110106

[14] Pindus DM , Mullis R , Lim LC , et al. Stroke survivors' and informal caregivers' experiences of primary care and community healthcare services—a systematic review and meta‐ethnography. PLoS One. 2018;13(2):e0192533. 10.17863/CAM.23117 29466383 PMC5821463

[15] The Norwegian Ministry of Health and Care Services . *The Health&Care21*. 2014. Accessed December 17, 2021.https://www.regjeringen.no/contentassets/8ab2fd5c4c7746dfb51e3f64cd4d71aa/helseomsorg21_strategi_web.pdf?id=2266705

[16] Helse‐ og omsorgsdepartementet . National Health and Care services Plan (2011‐2015). 2011.

[17] INVOLVE . What is public involvement in research? NIHR. November 12, 2020. Accessed June 20, 2021. https://www.invo.org.uk/find-out-more/%20what-is-public-involvement-in-research-2/

[18] Crowe S , Fenton M , Hall M , Cowan K , Chalmers I . Patients', clinicians' and the research communities' priorities for treatment research: there is an important mismatch. Res Involv Engagem. 2015;1(1):2. 10.1186/s40900-015-0003-x 29062491 PMC5598091

[19] Chalmers I , Bracken MB , Djulbegovic B , et al. How to increase value and reduce waste when research priorities are set. Lancet. 2014;383(9912):156‐165. 10.1016/S0140-6736(13)62229-1 24411644

[20] Viergever R , Olifson S , Ghaffar A , Terry R . A checklist for health research priority setting: nine common themes of good practice. Health Res Policy Syst. 2010;8(1):36. 10.1186/1478-4505-8-36 21159163 PMC3018439

[21] Greenhalgh T , Hinton L , Finlay T , et al. Frameworks for supporting patient and public involvement in research: systematic review and co‐design pilot. Health Expect. 2019;22(4):785‐801. 10.1111/hex.12888 31012259 PMC6737756

[22] Snow R , Crocker JC , Crowe S . Missed opportunities for impact in patient and carer involvement: a mixed methods case study of research priority setting. Res Involv Engagem. 2015;1(1):7. 10.1186/s40900-015-0007-6 29062496 PMC5611607

[23] Cowan K , Oliver S , Cowan K , eds. The James Lind Alliance Guidebook Version 8. 8th ed. National Institute for Health Research; 2018.

[24] Pollock A , St George B , Fenton M , Firkins L . Top 10 research priorities relating to life after stroke—consensus from stroke survivors, caregivers, and health professionals. Int J Stroke. 2014;9(3):313‐320. 10.1111/j.1747-4949.2012.00942.x 23227818

[25] Franklin S , Harhen D , Hayes M , Demos Mc Manus S , Pollock A . Top 10 research priorities relating to aphasia following stroke. Aphasiology. 2018;32(11):1388‐1395. 10.1080/02687038.2017.1417539

[26] Stroke Association . *Shaping stroke research to rebuild life—the Stroke Priority Setting Partnership results for investment*. July 2021. Accessed June 20, 2021. https://www.stroke.org.uk/sites/default/files/research/stroke_priority_setting_partnership_full_report.pdf

[27] Yoshida S . Approaches, tools and methods used for setting priorities in health research in the 21st century. J Glob Health. 2016;6(1):1‐10. 10.7189/jogh.06.010507 PMC4576459 26401271

[28] OsloMet . Brobyggersatsningen. OsloMet. Accessed May 27, 2021. https://www.oslomet.no/om/hv/fou/brobyggerprosjekt

[29] Akershus universitetssykehus . Ahus. Om oss. Accessed June 3, 2021. www.ahus.no

[30] Ormstad H , Jamtvedt G , Svege I , Crowe S . The Bridge Building Model: connecting evidence‐based practice, evidence‐based research, public involvement and needs led research. Res Involv Engagem. 2021;7(1):1‐77. 10.1186/s40900-021-00320-y 34717755 PMC8557598

[31] Trevelyan EG , Robinson N . Delphi methodology in health research: how to do it? Eur J Intergr Med. 2015;7(4):423‐428.

[32] Levelink M , Voigt‐Barbarowicz M , Brütt AL . Priorities of patients, caregivers and health‐care professionals for health research—a systematic review. Health Expect. 2020;23(5):992‐1006. 10.1111/hex.13090 32643854 PMC7696132

[33] Brett J , Staniszewska S , Mockford C , et al. A systematic review of the impact of patient and public involvement on service users, researchers and communities. Patient. 2014;7(4):387‐395. 10.1007/s40271-014-0065-0 25034612

[34] Hughes M , Duffy C . Public involvement in health and social sciences research: a concept analysis. Health Expect. 2018;21(6):1183‐1190. 10.1111/hex.12825 30159960 PMC6250854

[35] Tong A , Synnot A , Crowe S , et al. Reporting guideline for priority setting of health research (REPRISE). BMC Med Res Methodol. 2019;19(1):243. 10.1186/s12874-019-0889-3 31883517 PMC6935471

[36] Staniszewska S , Brett J , Simera I , et al. GRIPP2 reporting checklists: tools to improve reporting of patient and public involvement in research. BMJ. 2017;358:j3453. 10.1136/bmj.j3453 28768629 PMC5539518

[37] Braun V , Clarke V . Using thematic analysis in psychology. Qual Res Psychol. 2006;3(2):77‐101. 10.1191/1478088706qp063oa

[38] Oikarinen A , Kääriäinen M , Kyngäs H . A framework of counseling for patients with stroke in nursing: a narrative literature review. J Neurosci Nurs. 2014;46(5):E3‐E14. 10.1097/JNN.0000000000000079 25188689

[39] Clarke DJ . Nursing practice in stroke rehabilitation: systematic review and meta‐ethnography. J Clin Nurs. 2014;23:1201‐1226. 10.1111/jocn.12334 24102924

[40] Gonçalves‐Bradley D , Lannin N , Clemson L , Cameron I , Shepperd S . Discharge planning from hospital. Cochrane Database Syst Rev. 2016;1:CD000313. 10.1002/14651858.CD000313.pub5 PMC7073416 26816297

[41] Allison R , Shelling L , Dennett R , Ayers T , Evans P , Campbell J . The effectiveness of various models of primary care‐based follow‐up after stroke: a systematic review. Prim Health Care Res Dev. 2011;12(3):214‐222. 10.1017/S146342361100003X 21798119

[42] Siemonsma P , Döpp C , Alpay L , Tak E , Meeteren Nv , Chorus A . Determinants influencing the implementation of home‐based stroke rehabilitation: a systematic review. Disabil Rehabil. 2014;36(24):2019‐2030. 10.3109/09638288.2014.885091 24520957

[43] Miller KK , Lin SH , Neville M . From hospital to home to participation: a position paper on transition planning poststroke. Arch Phys Med Rehabil. 2019;100(6):1162‐1175. 10.1016/j.apmr.2018.10.017 30465739

[44] Fens M , Vluggen T , van Haastregt JC , Verbunt JA , Beusmans GH , van Heugten CM . Multidisciplinary care for stroke patients living in the community: a systematic review. J Rehabil Med. 2013;45(4):321‐330. 10.2340/16501977-1128 23546307

[45] Luker J , Murray C , Lynch E , Bernhardsson S , Shannon M , Bernhardt J . Carers' experiences, needs, and preferences during inpatient stroke rehabilitation: a systematic review of qualitative studies. Arch Phys Med Rehabil. 2017;98(9):1852‐1862. 10.1016/j.apmr.2017.02.024 28363703

[46] Smith TO , Pearson M , Pfeiffer K , Crotty M , Lamb SE . Caregiver interventions for adults discharged from the hospital: systematic review and meta‐analysis. J Am Geriatr Soc. 2019;67(9):1960‐1969. 10.1111/jgs.16048 31350918

[47] Lou S , Carstensen K , Jørgensen CR , Nielsen CP . Stroke patients' and informal carers' experiences with life after stroke: an overview of qualitative systematic reviews. Disabil Rehabil. 2017;39(3):301‐313. 10.3109/09638288.2016.1140836 26882958

[48] Dixon‐Woods M , Cavers D , Agarwal S , et al. Conducting a critical interpretive synthesis of the literature on access to healthcare by vulnerable groups. BMC Med Res Methodol. 2006;6(1):35. 10.1186/1471-2288-6-35 16872487 PMC1559637

[49] Forster A , Brown L , Smith J , et al. Information provision for stroke patients and their caregivers. Cochrane Database Syst Rev. 2012;11(11):CD001919. 10.1002/14651858.CD001919.pub3 23152210 PMC6544775

[50] Hesselink G , Vernooij‐Dassen M , Pijnenborg L , et al. Organizational culture: an important context for addressing and improving hospital to community patient discharge. Med Care. 2013;51(1):90‐98. 10.1097/MLR.0b013e31827632ec 23132202

[51] The Research Council of Norway . *Forskning og innovasjon i kommunesektoren‐FORKOMMUNEProgramplan*. 2017. Accessed December 16, 2021. https://www.forskningsradet.no/contentassets/5f3a8f57d212459a9917c72830ff59d6/programplan_forkommune_revidert_2019.pdf

[52] Magnussen S , Andfossen NB , Høiland GCL . *Kommunal medvirkning og samarbeid med forskningsmiljøer om innovasjon og forsknings i omsorgssektoren*. 2021. p. 23.

[53] Nygaard A , Halvorsrud L , Linnerud S , Grov EK , Bergland A . The James Lind Alliance process approach: scoping review. BMJ Open. 2019;9(8):e027473. 10.1136/bmjopen-2018-027473 PMC6720333 31473612

[54] Pollock A , St George B , Fenton M , Crowe S , Firkins L . Development of a new model to engage patients and clinicians in setting research priorities. J Health Serv Res Policy. 2014;19(1):12‐18. 10.1177/1355819613500665 24004532

[55] The James Lind Alliance Priority setting Partnerships . The JLA priority setting workshop online. James Lind Alliance. Accessed November 12, 2021.https://www.jla.nihr.ac.uk/jla-guidebook/chapter-8/the-jla-priority-setting-workshop-online.htm

[56] Boote JD , Dalgleish M , Freeman J , Jones Z , Miles M , Rodgers H . ‘But is it a question worth asking?’ A reflective case study describing how public involvement can lead to researchers' ideas being abandoned. Health Expect. 2014;17(3):440‐451. 10.1111/j.1369-7625.2012.00771.x 22646745 PMC5060724

